# Coexistence of monomorphic epitheliotropic intestinal T-cell lymphoma of the jejunum and bilateral adrenal pheochromocytomas: A case report

**DOI:** 10.1016/j.radcr.2025.09.021

**Published:** 2025-10-04

**Authors:** Tamaki Ichikawa, Makiko Kobayashi, Shunro Matsumoto, Rikio Suzuki, Sakura Tomita, Naoya Nakamura, Jun Hashimoto

**Affiliations:** a Department of Radiology, Tokai University School of Medicine, Isehara, Kanagawa, Japan; b Department of Radiology, Oita City Medical Association’s Almeida Hospital, Isehara, Kanagawa, Japan; c Department of Hematology/oncology, Tokai University School of Medicine, Isehara, Kanagawa, Japan; d Department of Pathology, Tokai University School of Medicine, Isehara, Kanagawa, Japan

**Keywords:** Monomorphic epitheliotropic intestinal T-cell lymphoma, PET-CT, Pheochromocytomas

## Abstract

Monomorphic epitheliotropic intestinal T-cell lymphoma (MEITL) is rare and aggressive form of intestinal lymphoma. We report a 52-year-old man with coexisting MEITL of the jejunum and bilateral adrenal pheochromocytomas. PET-CT revealed a small jejunal mass with intestinal wall thickening (SUVmax: 21) and bilateral adrenal lesions (SUVmax: 10). Diffusion-weighted whole-body imaging with background body signal suppression (DWIBS) showed restricted diffusion in the jejunal mass [Apparent diffusion coefficient (ADC): 0.71 × 10^−3^ cm/sec] and adrenal lesions (ADC: 1.2 × 10^−3^ cm/sec). Bilateral adrenal masses demonstrated I-123 MIBI uptake, consistent with pheochromocytomas. Simultaneous resection of the jejunal and bilateral adrenal tumors was performed. Pathological diagnoses were MEITL of the jejunum without lymph node involvement and benign bilateral adrenal pheochromocytomas. Despite prompt diagnosis and treatment, the patient died 9 months after initial presentation. This case underscores the importance of recognizing MEITL even when imaging findings appear mild and highlights the utility of PET-CT and DWIBS in its evaluation.

## Introduction

Enteropathy-associated T-cell lymphoma (EATL) is a rare extranodal T-cell lymphoma, accounting for approximately 0.1% of all malignant lymphomas and 0.5% of malignant lymphomas of the digestive tract [1,2]. EATL is subclassified into 2 types based on clinicopathological characteristics. Type I EATL is associated with celiac disease, an autoimmune disease triggered by gluten ingestion, and is predominantly reported in Northern European countries. In contrast, Type II EATL arises de novo and occurs globally, with a higher prevalence in Eastern countries [[1], [2], [3], [4]]. EATL type II was renamed monomorphic epitheliotropic intestinal T-cell lymphoma **(**MEITL) in the 2016 revision of the World Health Organization (WHO) classification [5]. MEITL is an extremely rare and aggressive lymphoma with a poor prognosis, often diagnosed when patients present with intestinal perforation or obstruction [6].

Herein, we report a rare case of a 52-year-old man with coexisting MEITL of the jejunum and bilateral adrenal pheochromocytomas, with a focus on the radiological and clinical features. To the best of our knowledge, this is the first reported case of coexisting MEITL of the jejunum and bilateral adrenal pheochromocytomas. Although CT findings of the jejunal lesion did not appear overtly malignant and prompt resection of the jejunum and adrenal lesions was performed, the patient died 9 months after the first medical examination.

## Case report

**Fig. 1:**
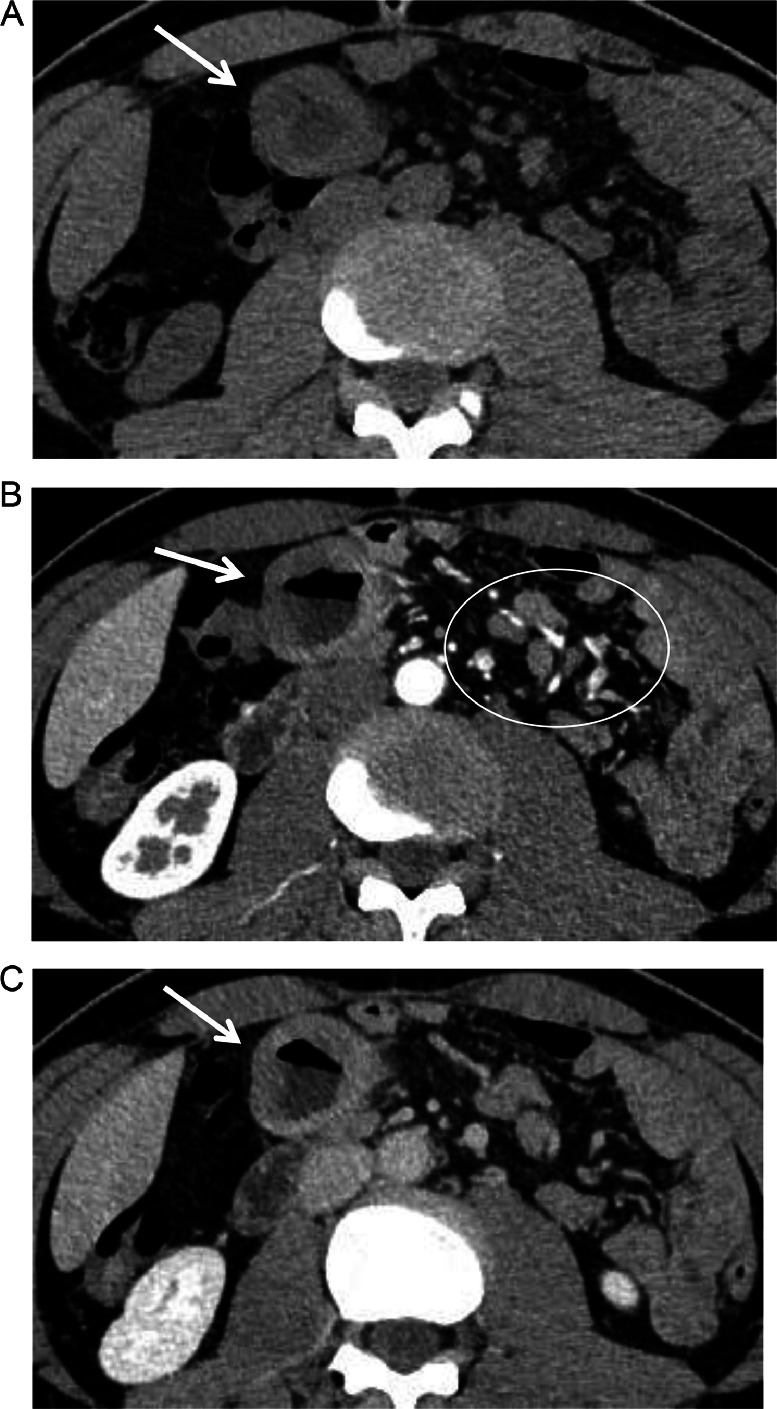
Dynamic CT images of the small intestine. Dynamic CT axial images (A-C) show a small aneurysmal dilatation of the jejunum with homogenous wall enhancement (white arrows). Mesenteric lymph node enlargement is observed (circle in B). A) Pre-contrast phase; B) early arterial phase; C) delayed phase.

**Fig. 2:**
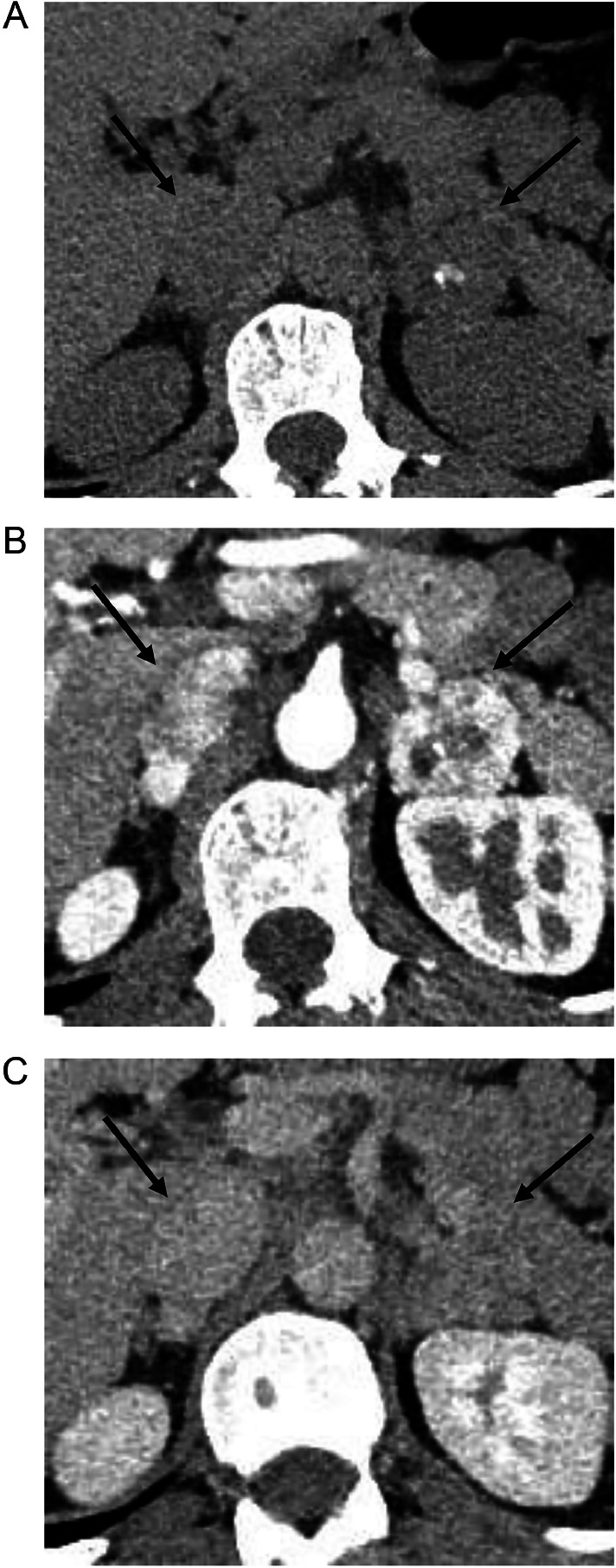
Dynamic CT images of the adrenal glands. Dynamic CT axial images show hyper-vascular masses in bilateral adrenal glands (black arrows) (A-C). Calcification is noted within the left adrenal solid mass (A). a) Pre-contrast phase; B) early phase; C) delayed phase.

**Fig. 3:**
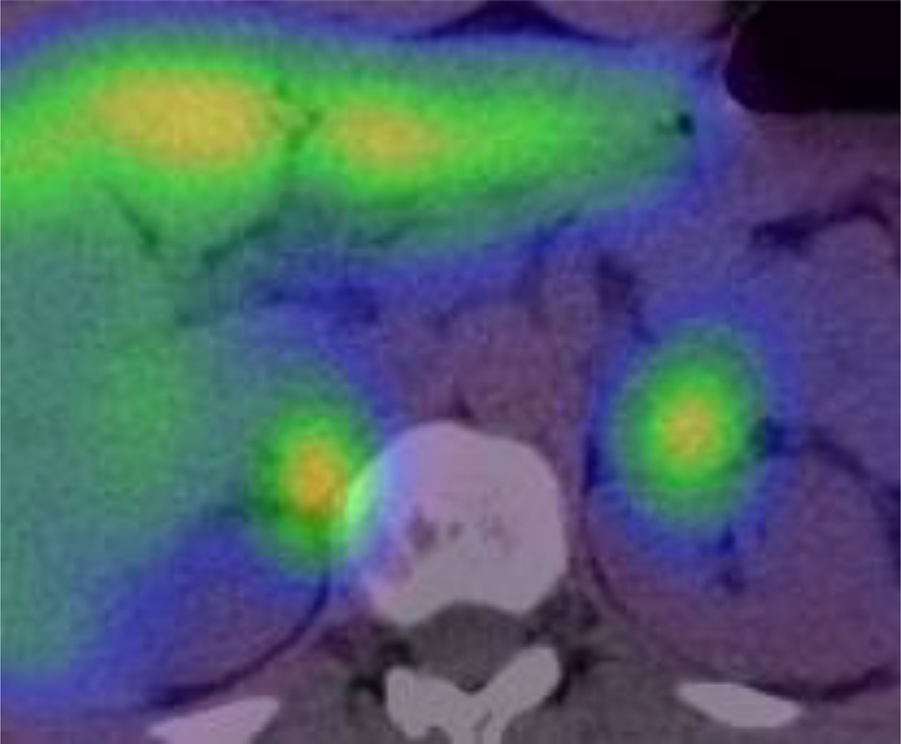
^131^I-MIBG scintigraphy. Both adrenal lesions exhibit apparent MIBG accumulations, consistent with bilateral pheochromocytomas.

**Fig. 4:**
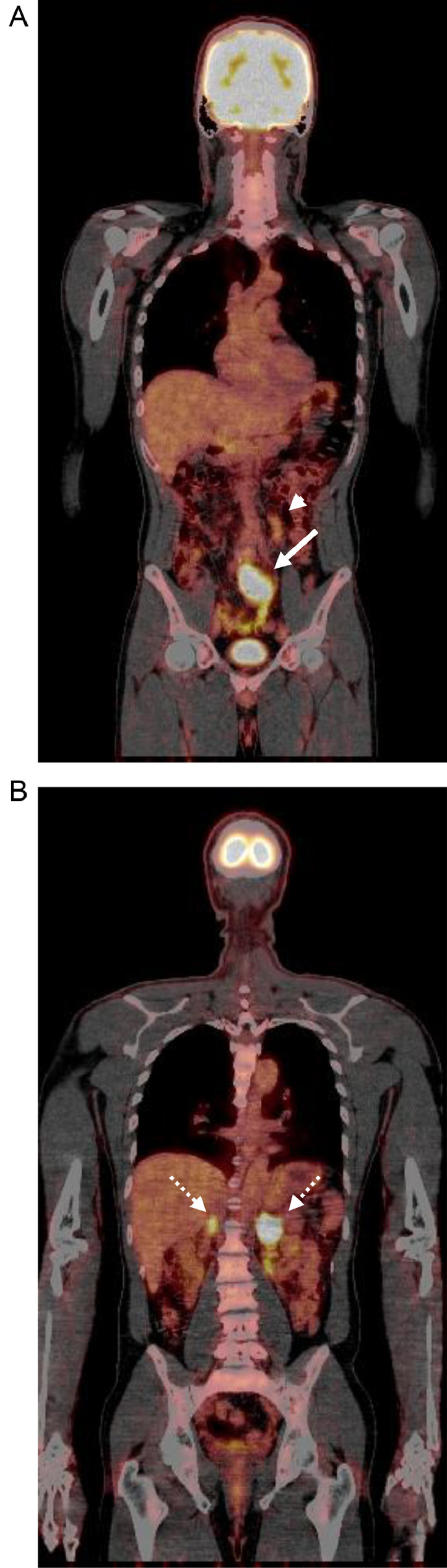
PET-CT images. Coronal PET-CT images show FDG uptake in the jejunal lesion (A: white arrow), a lymph node (A: arrowhead) and bilateral adrenal lesions (B: dotted arrows). SUV max values: jejunum = 10.3; lymph node = 3.3; left adrenal = 21.8; right adrenal = 15.

**Fig. 5:**
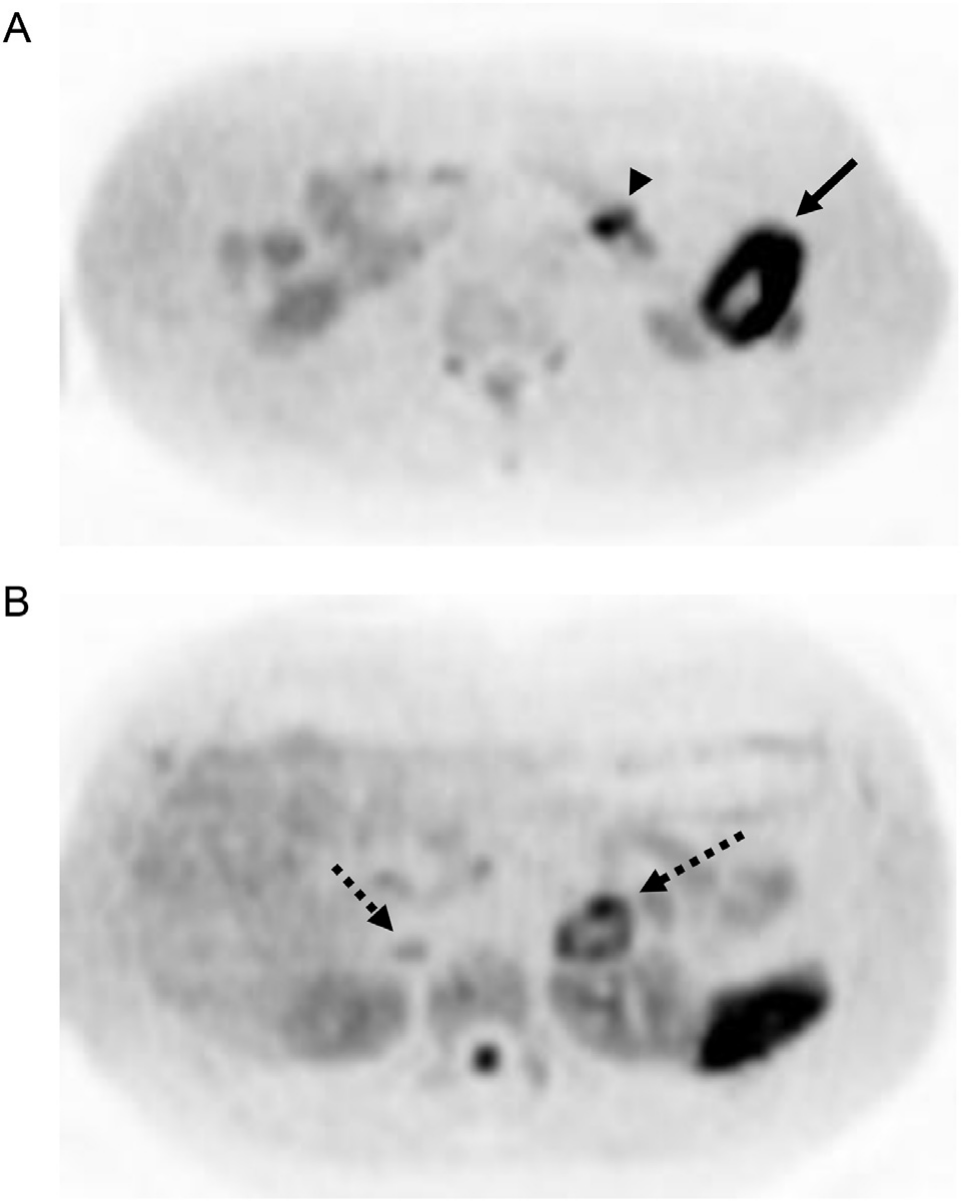
Diffusion-weighted whole-body imaging with background body signal suppression (DWIBS). Axial DWIBS images show restricted diffusion in the jejunal lesion (A: black arrow), a lymph node (A: arrowhead) and both adrenal lesions (B: dotted arrows). ADC values: jejunum = 0.71 × 10^−3^ cm/sec; left adrenal = 1.2 × 10^−3^ cm/sec.

**Fig. 6:**
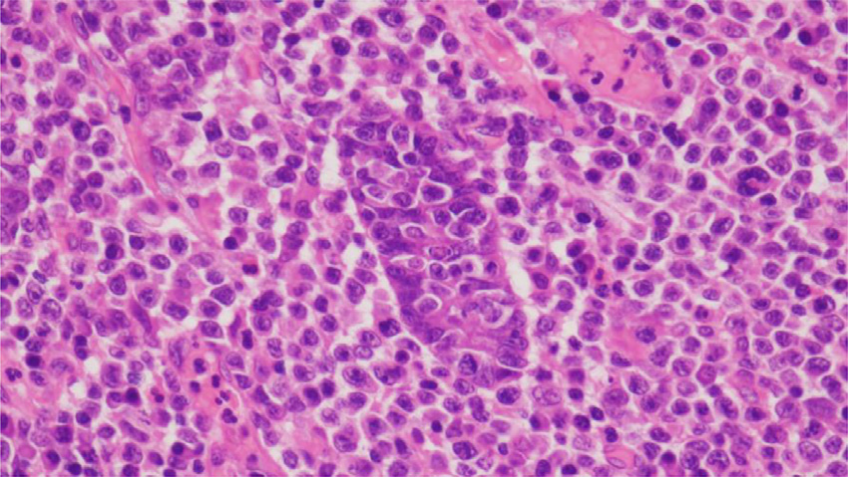
Histopathological images (H&E stain ×40). Hematoxylin and Eosin staining shows infiltration of medium- to large-sized atypical lymphocytes, with lymphoid cell infiltration throughout the intestinal wall.

**Fig. 7:**
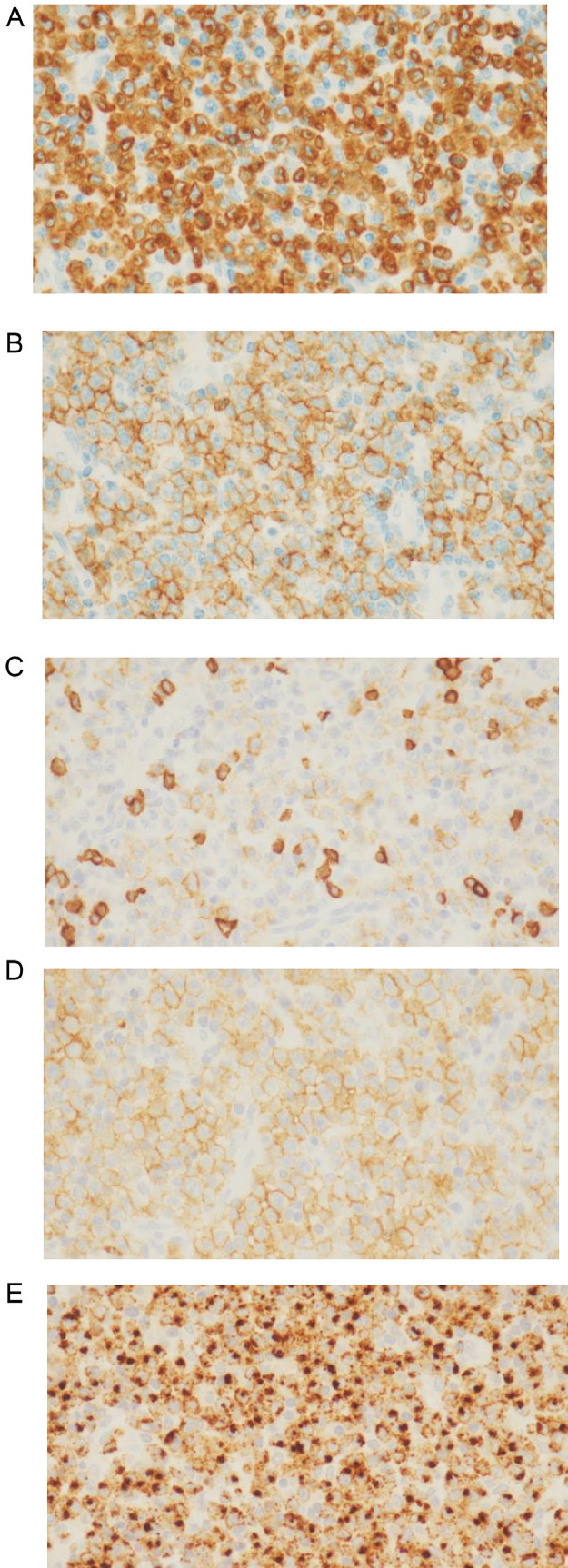
Immunohistochemical staining of tumor cells. Immunohistochemical analysis shows that the tumor cells are positive for CD3, CD8, CD20 (weak), CD56, and TIA-1. a) CD3; b) CD8; c) CD20; d) CD56; e) TIA-1.

A 52-year-old Thai male brain surgeon presented with abdominal pain, without any relevant family or past medical history. Abdominal ultrasonography revealed thickening of the small intestinal wall, prompting further evaluation with contrast-enhanced abdominal CT. Dynamic CT images (Fig. 1A–C) showed an infiltrating, circumferential enhancing mass with mild aneurysmal dilatation in the jejunum. Associated mesenteric lymph node enlargement was noted (Fig. 1B). Additionally, hyper-vascular masses were identified in bilateral adrenal glands (Fig. 2A–C), with the left adrenal mass showing calcification (Fig. 2a). Given the adrenal findings, pheochromocytomas were suspected. Iodine-131 metaiodobenzylguanidine **(**^131^I-MIBG) scintigraphy revealed apparent radiotracer accumulation in both adrenal lesions (Fig. 3), supporting the diagnosis. Due to suspicion of jejunal malignant lymphoma, PET-CT and diffusion-weighted whole-body imaging with background body signal suppression (DWIBS) were performed for further evaluation. PET-CT showed FDG uptake in the jejunal mass, both adrenal lesions, and mesenteric lymph nodes (Fig. 4). DWIBS demonstrated restricted diffusion in the same regions (Fig. 5). No other lesions were detected on PET-CT and DWIBS images. Laboratory data revealed normal IL-2 receptor **(**IL-2R) and lactate dehydrogenase (LDH) levels, and blood pressure was normal. However, total urinary metanephrine was slightly elevated at 1.21 mg/L. Pre-operative diagnoses were malignant lymphoma of the jejunum and bilateral adrenal pheochromocytomas. An alpha-adrenergic blocking agent was administered preoperatively. The patient underwent surgery 19 days after the initial medical examination, which included resection of both the jejunum and adrenal glands. A left adrenalectomy and right adrenal tumor resection were performed first, followed by partial resection of the jejunum and mesenteric lymph node dissection. Hematoxylin and Eosin (H&E) staining of the jejunal lesion showed infiltration of medium- to large-sized atypical lymphocytes throughout the intestinal wall (Fig. 6). Immunohistochemical staining showed that the tumor cells were positive for CD3, CD8, CD20 (weak), CD56, and TIA-1 (Fig. 7). The final pathological diagnoses were MEITL of the jejunum without lymph node involvement and benign bilateral adrenal pheochromocytomas.

**Fig. 8:**
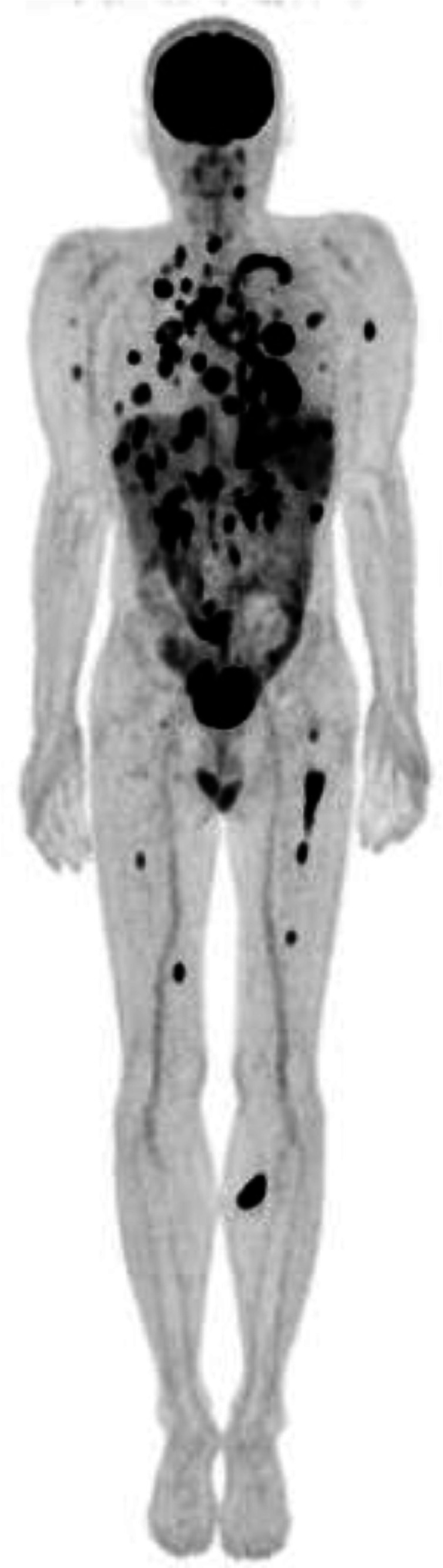
PET-CT image at 9 months after initial presentation. The maximum intensity projection (MIP) image shows multiple areas of FDG uptake, consistent with metastases.

The patient initially achieved remission after 6 cycles of CHOP chemotherapy. However, follow-up DWIBS imaging revealed multiple metastases (Fig. 8). He died 9 months after the first medical examination.

## Discussion

Enteropathy-associated T-cell lymphoma (EATL) is a rare primary T-cell lymphoma of the digestive tract. EATL is classified as either Type I, which is frequently associated with and thought to arise from celiac disease and is primarily observed in Northern Europe, and Type II, which occurs de novo and is distributed all over the world with predominance in Asia [[2], [3], [4], [5], [6]]. EATL type II was renamed MEITL in the 2016 revisions to the World Health Organization classification [5]. Approximately 300 cases of MEITL have been reported worldwide to date [7]. MEITL arises from the malignant proliferation of intraepithelial lymphocytes, with a mean age at diagnosis of approximately 60 years and a male-to-female ratio of approximately 2:1 [7]. The small intestine, particularly the proximal jejunum, is the most commonly affected site [8,9]. Early clinical symptoms are often insidious and nonspecific, including abdominal pain, bowel dilatation, or other vague gastrointestinal complaints [7,9,10]. As a result, most patients are diagnosed at an advanced stage of the disease, often with serious complications such as intestinal perforation or small bowel obstruction [6,[9], [10], [11], [12]]. The prognosis remains poor, with a median survival time of 7 months and a 1-year overall survival rate of only 36% [7,10].

The small intestinal manifestations of MEITL include multiple ulcerative lesions and mucosal edema, often without distinct tumor formation [6]. However, Kitano et al. reported that MEITL tends to form protruding masses, whereas EATL more commonly presents with ulcerating nodules, plaques, and strictures [11].

Histologically, METEL is characterized by small to medium-sized monomorphic lymphoid cells with diffuse infiltration throughout the intestinal wall [2,5]. Immunohistochemically, tumor cells are usually positive for CD3, CD8, CD56, TIA-1, granzyme B, and perforin, and negative for CD4 and CD5, consistent with the findings in our patient [5,6,8,13,14].

The genomic profile of MEITL has been previously reported, with almost all cases harboring deleterious mutation(s) and/or deletions in the *SETD2* gene [[13], [14], [15]]. Other frequently mutated genes include *STAT5B, JAK3, TP53, JAK1, BCOR*, and *ATM* [15]. The genetic profile of our patient was not available.

On MRI, MEITL typically exhibits iso- or hypointense signals on T2-weighted imaging (T2WI) and restricted diffusion on diffusion-weighted imaging, which is attributed to the high cellularity of the tumor and reduced extracellular fluid content [7]. In the present case, MEITL lesions also exhibited restricted diffusion on DIWBS. On contrast-enhanced imaging, MEITL typically shows delayed and homogeneous enhancement [7], a feature suggestive of the absence of necrosis.

Several authors have reported the PET-CT findings of MEITL, with lesions demonstrating increased FDG uptake (SUVmax: 4.0–14.8), as seen in our patient [7,12,16]. On PET-CT, MEITL must be differentiated from malignancies, such as EATL and aggressive B-cell lymphomas, as well as benign conditions like inflammatory bowel disease [7]. EATL typically appears as a hypermetabolic lesion with a high SUVmax value, against a background of diffuse FDG accumulation of mild intensity, reflecting refractory coeliac disease [7]. B-cell lymphomas usually present with markedly higher SUVmax values compared to MEITL [12]. Inflammatory bowel disease manifests as segmental bowel wall thickening and FDG uptake, but typically without intestinal dilatation, and is confined to specific sites [12]. Zhang et al. reported a case of MEITL with extensive involvement of the entire digestive tract, liver, and mesenteric lymph nodes on PET-CT [12]. In our patient, the initial PET-CT revealed FDG uptake in the mesenteric lymph nodes, although no pathological involvement was confirmed in these nodes. The end-stage PET-CT showed multiple intense FDG-avid metastases, indicating widespread disease progression (Fig. 8).

MEITL has been reported to metastasize to the lungs, liver, central nervous system, and bone marrow [7]. Due to its rarity, there are no standard treatment guidelines for MEITL [7]. In previous reports, cyclophosphamide, doxorubicin, vincristine, and prednisone were the most used chemotherapeutic agents [7,10,12]. However, anthracycline-containing chemotherapy has shown limited efficacy in MEITL patients [10,11].

The coexistence of MEITL and bilateral adrenal pheochromocytomas in our patient appeared to be a coincidental finding. As noted in previous studies, early diagnosis of MEITL prior to intestinal perforation or obstruction is critical [6,10,12]. In our patient, adrenal pheochromocytomas were diagnosed by ¹³¹I-MIBG scintigraphy, and an alpha-adrenergic blocking agent was administered for 2 weeks. Surgical treatment of both the jejunal and adrenal lesions was performed 19 days after the first medical examination. The patient, a medical doctor, was able to understand and accept the diagnostic process without requiring a pre-operative biopsy. On contrast-enhanced CT, the jejunal MEITL lesion appeared as a small aneurysmal dilated lesion, which did not initially suggest high malignancy. Aneurysmal dilation is a recognized radiologic feature of intestinal lymphoma, characterized by a circumferential infiltrative lesion with luminal enlargement due to tumor infiltration of the muscularis propria, resulting in disruption of the autonomic nerve plexus [17]. MRI serves as a valuable tool for detecting malignant MEITL lesions, whereas 18F-FDG PET-CT aids in tumor staging and assessing treatment efficacy [10,11]. In our patient, both the intestinal and lymph node lesions exhibited limited diffusion on DWBIS with similar apparent diffusion coefficient **(**ADC) values and high FDG uptake on PET-CT. These findings were consistent with MEITL. However, the SUVmax of the mesenteric lymph node (3.3) was lower than that of the jejunal lesion (10.3), and histopathological analysis confirmed no MEITL involvement in the lymph node. The extremely poor prognosis of MEITL is largely attributed to delayed diagnosis and the absence of effective targeted therapies [12]. In this case, despite a prompt diagnosis and timely surgical intervention, the patient died 9 months after the initial presentation.

Radiologists should pay close attention to the radiologic features of MEITL and maintain a high index of suspicion for malignant lymphoma, even when radiologic findings do not appear overtly aggressive. Early recognition is essential for timely diagnosis and treatment. Given that MEITL is a rare but aggressive disease with a poor prognosis, awareness and understanding of its clinical and radiologic presentation are crucial for both doctors and patients.

## Conclusion

We reported the first documented case of coexisting MEITL of the jejunum and bilateral adrenal pheochromocytomas, with a focus on radiological and clinical findings. PET-CT and DWBIS proved to be valuable tools for the detection, staging, and treatment monitoring of MEITL. Despite occasionally subtle radiologic appearances, MEITL is an aggressive malignancy with a poor prognosis.

## Patient consent

Informed consent for publication of their case was obtained from the patient.
